# Alignment of patient‐centredness definitions with real‐life patient and clinician experiences: A qualitative study

**DOI:** 10.1111/hex.13674

**Published:** 2022-12-03

**Authors:** Julie Babione, Dilshaan Panjwani, Sydney Murphy, Jenny Kelly, Jessica Van Dyke, Maria Santana, Jaime Kaufman, Peter Sargious, Doreen Rabi

**Affiliations:** ^1^ Department of Medicine, Cumming School of Medicine, O'Brien Institute for Public Health University of Calgary Calgary Alberta Canada; ^2^ Departments of Paediatrics and Community Health Sciences, Cumming School of Medicine, O'Brien Institute for Public Health University of Calgary Calgary Alberta Canada; ^3^ Department of Medicine, Cumming School of Medicine, W21C Research and Innovation Centre, O'Brien Institute for Public Health University of Calgary Calgary Alberta Canada

**Keywords:** patient and public involvement, patient empowerment, patient experience, patient‐centred care

## Abstract

**Introduction:**

Patient‐centred care (PCC) has come to the forefront for many institutions, funding agencies and clinicians, and is integrated into care. Does a disconnect in understanding still exist between patients, healthcare organizations and clinicians in what PCC means and how outstanding issues might be addressed?

**Methods:**

We conducted interviews and focus groups with self‐reported chronic care patients and clinicians providing care to these patients exploring PCC experiences, expectations and practices. These data were initially analysed using inductive thematic analysis. This paper reports on the findings of a secondary analysis examining the alignment between patients and clinicians on five key predetermined dimensions of PCC.

**Results:**

Eighteen patients participated, representing a range of chronic conditions. Thirty‐eight clinicians participated. One thousand and three hundred patient and 1800 clinician codes were identified and grouped into 5 main topics with 140 unique themes (patients) and 9 main topics with 54 unique themes (clinicians). A total of 166 quotes (patient = 93, clinician = 73) were identified for this PCC definition alignment analysis. Partial or complete alignment of patient and clinician perspectives was seen on most dimensions. Key disconnects were observed in patient involvement, patient empowerment and clinician–patient communication. Only 18% of patients reported experiencing patient‐centred communication, whereas 57% of clinicians reported using patient‐focused communication approaches.

**Conclusion:**

Overall, study patients and clinicians endorse that many PCC elements occur. This study highlights key differences between patients and clinicians, suggesting persistent challenges. Clinician participants relayed their PCC approaches of informing and educating patients; however, patients often perceive these approaches as didactic, role‐diminishing and noncollaborative. Collaborative approaches, such as shared decision‐making, hold promise to bridge persistent PCC gaps and should be integrated into medical education programmes.

**Patient or Public Contribution:**

This project was conceived and executed with a co‐design approach wherein patients with chronic conditions who are trained in research (i.e., see descriptions of Patient and Community Engagement Research in the text) were involved in all stages of the research project alongside other researchers on the project team. Healthcare providers were involved as participants and as principal investigators in the project.

## 1 INTRODUCTION

The Institute of Medicine defines ‘patient‐centred care’ (PCC) as ‘care that is respectful of and responsive to the preferences, needs, and values of the patient’. The Wagner Chronic Care Model^1^, ^2^ has been the template for care provision for over two decades, with significant investment in PCC at policy and mandate levels,^3^, ^4^, ^5^ through patient advocacy^6^ and in performance measures.^7^, ^8^ In Canada, there is a proliferation of primary care teams and medical homes that provide PCC to diverse patients. While evidence suggests these care models (with particular emphasis on interprofessional collaboration and care integration) effect positive clinical outcomes,^9^, ^10^, ^11^, ^12^ it is unclear whether the patient's care experience is significantly improved, or tangibly different, than with traditional models.

The importance of the patient and their lived experience in informing care is recognized by healthcare organizations and research funding agencies that prioritize patient inclusion and partnership.^13^, ^14^, ^15^, ^16^ Clinicians may see PCC as a service delivery structure that better supports patients' needs. Concurrently, patients may reasonably expect PCC to centre care on their experience of a condition, and formally include them in care processes and decisions that relate to them. A shared understanding of PCC's meaning is fundamental to achieving health care centred on patients, but the degree to which the delivery of PCC at the clinician level and the experience of PCC at the individual level are not extensively characterized.

Recognizing this potential disconnect between the agents and the objects of PCC, we conducted a secondary content analysis of patient and clinician narratives using principles of natural language processing^17^ to determine (1) whether contemporary care experiences are patient‐centred and (2) how patient and clinician perceptions of PCC align.

## 2 METHODS

Our team collected patient and clinician narratives as part of focus groups and interviews in the context of developing a patient‐centred planning tool for adults with multiple chronic health conditions. We conducted a secondary content analysis exploring PCC experiences from two distinct perspectives: patients and clinicians. Using a conceptual model of PCC^18^, ^19^, ^20^ (detailed below) we identified keywords and themes to capture experiences of PCC in patient and clinician narratives.

### 2.1 Focus groups and interviews with patients

Patients were recruited through the Patient and Community Engagement Research (PaCER) programme at the University of Calgary.^21^ PaCER researchers are patients trained in research methodologies (i.e., interviews, focus groups and surveys). PaCER's research is iterative, with three distinct data collection and analysis phases: Set, Collect and Reflect.^22^ We recruited study participants through outpatient speciality clinics and through existing networks.

Within the PaCER patient ‘Collect’ phase, data were captured through audio recordings, flip charts and process recording notes. Audio recordings were transcribed and analysed, deducing themes that informed the last (Reflect) phase, which explored participants' experiences of self‐management. Supplementary (Collect phase) interviews were added until no new themes emerged.

### 2.2 Focus groups and interviews with clinicians

This research team conducted semistructured interviews and focus groups with clinicians, following a guideline script that explored the understanding of patient‐centredness, how they involve patients in care planning, potential digital platform information elements and perceived general barriers and facilitators to digital platform uptake. With the script as a guideline, researchers were able to expand and elaborate, exploring topics, concepts, examples and responses to patient content as they emerged in the same and in subsequent sessions. Local primary care networks facilitated recruitment through targeted invitations supplemented by existing networks and sessions were audio‐recorded.

### 2.3 Participant data analysis

All sessions were anonymized, transcribed and verified. Four researchers (J. B., J. K., S. M., J. V. D.) including a social sciences expert in qualitative research methods (J. K.) conducted the inductive thematic analysis.^23^, ^24^ Researchers independently reviewed and coded transcripts in pairs, each team delegated half the transcripts. Codes were then reconciled through consensus discussions.

### 2.4 Alignment analysis

In parallel, another researcher (D. P.) used the analysis to identify specific quotes that aligned with the top five dimensions of PCC. Table 1 shows these dimensions that were derived following systematic review, consensus approach and selected as representative of a rigorous PCC concept synthesis.^19^

**Table 1:** Dimensions of PCC^18^

Dimension	Definition
Patient as a unique person	Each patient's individual needs, preferences, values, feelings, concerns, ideas and expectations as well as exploring both the patient's disease and illness experience, the impact on functions (e.g., the patient's idea of how the illness affects his or her daily life; effects of the illness on the patient and his or her family), and his or her individual explanatory model. This also entails providing care that is tailored to each specific patient.
Clinician–patient communication	Many aspects of how we communicate in a patient‐centred manner are included in the definitions of patient‐centredness. They include general communication skills, (e.g., setting the stage, setting an agenda, prioritizing the patient's problems). A broad range of verbal and nonverbal behaviour can be used to engage in patient‐centred communication (e.g., using open‐ended questions, summarizing important information, asking the patient to repeat, making eye contact, nodding).
Patient information	This dimension highlights the importance of sharing knowledge and information reciprocally between the clinician and the patient. The clinician should give tailored information (regarding all aspects of care from prevention to treatment, as well as information on how to access medical, psychosocial, physical and financial support) while eliciting and respecting the patient's information needs and preferences. Some definitions also described the provision of informational resources and tools (e.g., audio records of consultations, multimedia resources, information brochures). Furthermore, the patient should be encouraged to share information (e.g., regarding symptoms and concerns).
Patient involvement in care	A prominent dimension often described in the literature on patient‐centeredness is the patient's active involvement in care. While older publications use terms like ‘informed consent’ or ‘sharing power and responsibility,’ more recent publications define in more detail the importance of encouraging the patient to participate actively in the consultation and of engaging the patient in the decision making regarding his or her own health (shared decision making). The importance of helping the patient in making informed choices is highlighted in many definitions. This includes respecting the patient's preferences for involvement as well as encouraging the patient's feedback on care (e.g., using patient surveys).
Patient empowerment	‘…by acknowledging the patients' perceived ability to self‐manage important aspects of his or her illness, activating and encouraging the patient to take responsibility to solve health related problems and to take actions to improve his or her health and becoming an expert regarding the management of his or her health condition. This also entails supporting the patient's autonomy by offering educational programs, patient activation and health promotion interventions’.

*Note*: Content reproduced with permission under the Creative Commons Attribution License.

Abbreviation: PCC, patient‐centred care.

Two researchers (D. P. and J. B.) independently coded quotes within dimensions according to *concept presence* and *concept concordance*. For concept presence, quotes were evaluated whether they *referred* to a specific dimension. For concept concordance, quotes were examined for *alignment with* the dimension.

For example, for the dimension ‘patient as a unique person’, concept presence was (‘recognition of each patient's uniqueness—individual needs, preferences, values, feelings, beliefs, concerns, ideas, expectations’) and concordance ‘the patient *is* a unique person’. If a quote spoke about patient uniqueness *and* that the patient *is* unique, both criteria are met. Quotes were colour‐coded as:

- *Green* (both concept presence and concordance match), illustrating the selected quote was present and in complete alignment with the dimension.
- *Yellow* (concept presence *or* concordance match), illustrating that either the quote was relevant for the dimension *or* the viewpoint was in alignment.
- Red (neither concept presence nor concordance match). This might occur if the first researcher (D. P.) reconsidered the quote, or the second researcher (J. B.) disagreed that the quote was sufficiently applied.

Coding consensus was established on all included quotes and explicit comparisons between patient and clinician alignment quantified and visualized.

## 3 RESULTS

### 3.1 Focus groups and interviews

Eighteen patients participated, representing a range of chronic conditions, including cancers, diabetes, liver failure, leukaemia, bone marrow transplant, heart problems, scleroderma, chronic obstructive pulmonary disease, Hashimoto's thyroiditis, arthritis, depression, bipolar disorder and anxiety. Table 2 summarizes these demographics. Thirty‐eight clinicians participated in interviews or focus groups with representation as shown in Table 3.

**Table 2:** Demographic descriptors of patient participants

	*N* (%)
Basic demographic	
Sex (female)	11 (64.7)
Age	60.94 (12.3)
Education level	
High school diploma	3 (17.6)
College or university	14 (82.4)
Working status	
Full time/part time	4 (23.5)
Retired	7 (41.2)
Disability/sick Leave	5 (29.4)
Unemployed	1 (5.9)
Ethnicity	
Asian	1 (5.9)
East Indian	1 (5.9)
Caucasian	15 (88.2)
Family physician	
Yes	16 (94.1)
Self‐reported health status	
Very good	3 (17.6)
Good	7 (41.2)
Fair	5 (29.4)
Poor	2 (11.8)
Other descriptive demographics	
Number of medical conditions	3.59 (1.5)
Average number of healthcare providers on patient's team	3.82 (2.5)
Time managing health conditions (hours/week)	25.45 (46.97)

**Table 3:** Clinician participant demographics

Clinician type	# Participants
Physician	3
Specialist (Geriatric Medicine, Internal Medicine (2), Respirology, Neurology, Infectious Disease, Nephrology)	7
Pharmacist	5
Nurse	11
Social work	3
Dietician	1
Other (Kinesiologist, Medical Office Assistant, Behavioural Health Consultant (2), Patient Flow Coordinator, Clinic Manager, Unit Manager, Allied Health Manager)	8
Urban/rural	30/8

### 3.2 Primary participant data analysis

One thousand and three hundred patient and 1800 clinician codes were identified. Once both data sets were coded, two researchers (S. H. and J. K.) grouped codes into 5 main topics with 140 unique themes (patients) and 9 main topics with 54 unique themes (clinicians).

### 3.3 PCC definition alignment analysis

A total of 166 quotes (patient = 93, clinician = 73) were identified for this PCC definition alignment analysis. The distribution of quotes across themes is summarized in Table 4.

**Table 4:** Number of patient, clinician and total quotes as included in alignment analysis

Dimension	Patient quotes	Clinician quotes	Total quotes
Patient as a unique person	20	19	39
Clinician–patient communication	21	14	35
Patient information	18	15	33
Patient involvement in care	17	10	27
Patient empowerment	17	15	32
**Total**	**93**	**73**	**166**

**Figure 1:**
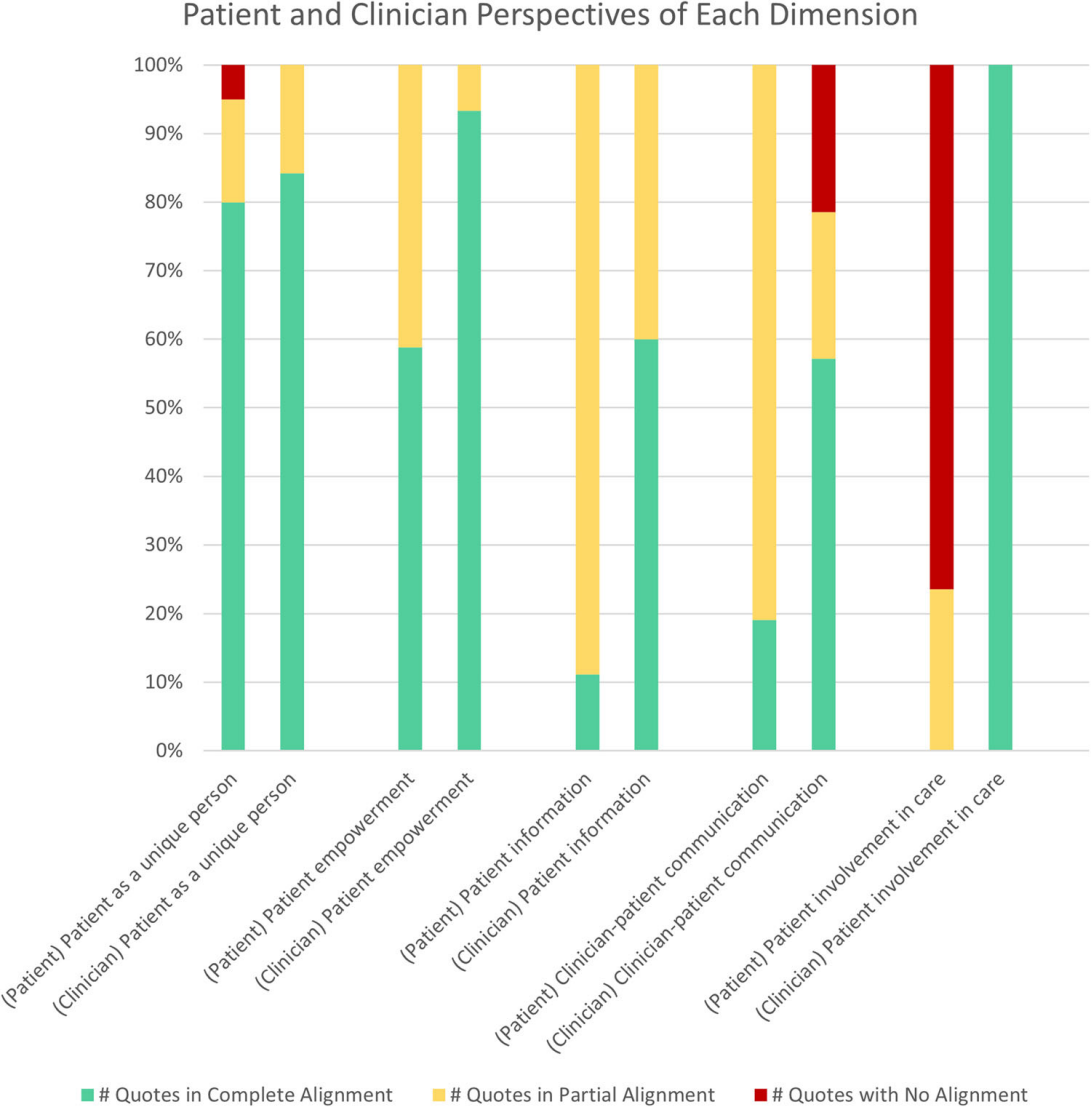
Patient and clinician perspectives of the top five dimensions, shown as percentages

As Figure 1 shows, patient and clinician narratives suggested that many elements of PCC are being achieved across most dimensions. Table 5 captures key example quotes for each dimension referenced in the text.

**Table 5:** Sample patient and clinician quotes included in this secondary alignment analysis

Dimension	Patient quotes	Clinician quotes
Patient as a unique person	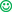 ‘Having been depressed before and having been worsely depressed, being terrified about where that spiral went, I actually did a lot of self‐controlling and saying, “I'm not going there again” and how do you not go there again? You continue to fight. So, from a previous experience had developed a whole array of tools’.	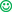 ‘Well, I'm seeing someone, we made some really good diet and exercise goals. Her goals, they're doable, we checked you know, confidence, importance, readiness and from those found some more barriers and then kind of did a plan B…’.
	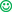 ‘And when I got back to my family a couple of weeks ago, one of my cousins said to me “You look just like five years ago. You haven't changed, or you don't act different. You're not marked,” she said’.	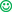 ‘The dynamic has switched here. It's easy for me to sit here in my seat and say you need to do a, b, c, d, but you need to tell me what you are able to do and willing to do’.
Patient empowerment	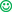 ‘…you have to be an advocate for yourself as much as possible because if they say, “Don't call us, we'll call you,” I never take that advice. I call and make sure I'm told’.	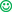 ‘I try to encourage them to like be involved as actively as they can, so whether that is voicing their concerns, or just kind of updating people as things go along so we know, and to provide us enough information so we know where we're coming from’.
	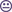 ‘Of course, the first thing he said to me, when he got my results back—**he eventually did agree to do them because I said “well if you're not going to run these tests for me, I will find someone who will”** because I knew a lot of the things were happening to me and I didn't like what was happening to me. So eventually he did agree to run them and the first thing he said was “Are you ok?” I didn't even register on the scale on this one type of test he did’.	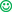 ‘And yes, we do involve our patients […] we do the congestive heart failure teaching, the diabetes teaching, the COPD teaching. If I have the opportunity to do that. […] Very basic, but putting it back to them, that they need to do that for themselves’.
Patient information	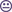 ‘I had my huge meltdown and I actually had been told the plan. And it was just around the very beginning and the plan was that one induction and two consolidation chemos. But I didn't hear that I had two consolidation chemos. I thought I only had one. So, when I went in there, I found out that I had two and that was meltdown. **And I actually had been told that, I was just so sick that I hadn't got it**’.	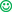 ‘I'm imagining something they can interpret versus like our actual care plan that we always see where you need to understand the medical jargon and understand what everything means’.
	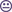 ‘You have reached a point where you are **not quite sure what was just said**, you don't remember what drugs you were supposed to take etc. **And there are a number of breakdowns in that interaction**’.	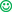 ‘I do a lot of discharge instructions sometimes for the patient because you can see that sometimes they don't understand or even though they are given a discharge summary with everything in there, they are still overwhelmed by everything. So, I'll just give the 1, 2, 3, 4, and that's after talking to the residents if they're going to do discharge instructions’.
Patient–clinician communication	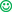 ‘I think it's incredibly important. I would say […] so this is what is going to happen now. And then after that somebody is going to discuss with you what the next step is, and this will take this many days. And then you are going to talk to this person and this person and then they will determine what the next time is that you're looking at’.	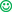 ‘I think one of the things that I have seen is the knowledge, the actual ability to understand the implications of the medical perspective. A lot of the information that a team will talk about is very technical […] you have to simplify it quite a bit so even if you were to attempt to … I don't know if patients and families would ever fully understand the whole picture’.
	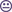 ‘It's one thing to go to the doctor and he knows and does everything, and the nurses do everything, **but they don't really communicate with you and tell you…**’.	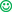 ‘Or taking that with them to go home and how they can further educate themselves. So, I educate them why I get them to do deep breathing to prevent pneumonia, to move fluids around, and I call it chest physio. And I explain to them exactly why I get them to do that’.
Patient involvement in care	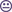 ‘Now I've got other health issues […] and I tell a doctor, a new system of doctors something, and they say, “oh no, that's nothing” and **they don't believe me**. You know all that credibility I had built up at the [other] clinic, in [this] suddenly I'm stupid, and you know that's not true right. **I find that really insulting**’.	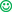 ‘I think […] to keep putting it back to the patients so what can you do, and how are you going to make this work, and just keep working with them on it until they start to take it on and start to get the hang of it’.
	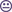 ‘Given what I have figured out from the literature and stuff, **it feels like the doctors I was dealing with were very—this is the way I have done it for twenty years, why should I change what I'm doing**?’	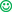 ‘…this only applies to some of our patients, sometimes some of the stuff about patient accountability and patient responsibility for their own health. And how do we draw them in as a partner rather than a victim of health care’.

*Note*: Quotes deemed to be in perfect alignment (concordance) with the dimension are precluded with a green 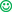, and those in partial alignment (partial discordance) with light orange 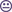. Selective text bolding within quotes highlights those elements that signalled the partial classification of those quotes.

Abbreviation: COPD, chronic obstructive pulmonary disease.

#### 3.3.1 Patient as a unique person

Patients and clinicians aligned well on the ‘patient as a unique person’ dimension, with over 80% of extracted quotes explicitly highlighting the importance of patients being recognized as individuals.

#### 3.3.2 Patient empowerment

There was also similar alignment on patient empowerment with 58% of patient quotes expressing feelings of empowerment, and 94% of clinician quotes supporting patient empowerment. Despite no overt disagreement on this dimension, patient and clinician quotes suggest interpretational differences and thus discordance in understanding between groups. Often, patient–participants likened empowerment to self‐advocacy and the ability to self‐determine care and outcomes, as in:

I've become very resourceful so I think for me, managing it if I know where my resources are, I know what the treatments are; I'm very good at asking the questions and knowing what is the best way to manage it. (Patient) 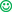

Some patients often viewed empowerment as a desire for emancipation from conventional practices that limit their role in accessing health information or prevent autonomous decision‐making. Conversely, clinicians often viewed patient empowerment as engagement in self‐care, being ‘educated’, a patient's ability to self‐manage or monitor or relaying information between clinicians. One example quote that speaks to this is:

You get them to go to the kiosk to look up information and follow up with it. They come in with diabetes but they also have hypertension so we get them to watch a video on why you want to monitor at home and why it's important to bring those readings to the doctor…. (Clinician) 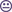

#### 3.3.3 Patient information

On this dimension, patient and clinician quotes suggest partial or complete alignment. Patient quotes were often partially aligned, expressing a desire for more reciprocal or situation‐appropriate information sharing. They also expressed a desire for better access to their health information.

#### 3.3.4 Clinician–patient communication

Only 18% of patients reported experiencing patient‐centred communication, whereas 57% of clinicians reported using patient‐focused communication approaches. Notably, 22% of extracted clinician quotes suggested that a patient‐centred communication dimension was *not* being achieved. Patients often focussed on needing information about ‘next steps’ and having their concerns/questions addressed, as in:

It's one thing to go to the doctor and he knows and does everything, and the nurses do everything, but they don't really communicate with you. (Patient) 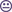

One patient expressed frustration and stress in recounting experiences awaiting direction. In contrast, clinicians expressed feeling successful at communicating with their patients. Most quotes spoke of clinicians simplifying medical explanations or ‘educating’ their patients, as in:

Or taking that with them to go home and how they can further educate themselves. So I educate them why I get them to do deep breathing to prevent pneumonia, to move fluids around, and I call it chest physio. (Clinician) 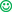

#### 3.3.5 Patient involvement in care

Patients and clinicians had strikingly different perceptions within this dimension. Most patients felt uninvolved, while interviewed clinicians expressed confidence in patient involvement. Clinicians spoke of attempts to engage patients in self‐care; however, patients felt their involvement was often prescribed or lacked autonomy. Patients also felt an undesirable off‐loading of care duties, making their ‘involvement’ burdensome with care workload concerns often going unheard. One patient participant recounted trying to engage in care decision‐making felt their efforts to self‐educate and inform care discussions were dismissed. Meanwhile, clinician participants saw patient involvement very differently—as executing prescribed self‐management plans, as in:

…they are the care giver for their husband or wife at home and its really difficult for them to get out. Really all I need to know is […] what have you done, what are your blood sugar numbers, how have you been adjusting your insulin and we just need to do some tweaking…. (Clinician) 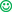

Sometimes, clinicians indicated trying to encourage patients to actively take responsibility for their own care. In other cases, clinicians indicated a desire to partner with their patients.

## 4 DISCUSSION

This study examined how patients' and clinicians' PCC experiences aligned across five dimensions considered integral to this care model and revealed important differences between patient and clinician perspectives and understanding of the dimensions, This suggests that there are several ongoing challenges in patient‐clinician care collaboration. For example, the dimensions of patient empowerment and patient involvement in their care *are* difficult to distinguish and can easily be misinterpreted. Clinician participants relayed their PCC approaches of informing and educating patients; however, patients often perceive these approaches as didactic, role‐diminishing and noncollaborative. If PCC is defined as being respectful and responsive to patients' preferences, values and needs, then reciprocal information sharing is integral for clinicians to fully understand each individual patient.

Our findings around unaligned PCC dimensions are consistent with prior research that patients feel insufficiently engaged or that the approach misses the target.^25^ Practices such as shared decision‐making (SDM) have been proposed to address these concerns. SDM is an approach where patients and clinicians collaboratively create personalized care decisions for each patient based on a shared review of relevant clinical and experiential data to the care decision and define a path that makes intellectual, emotional and practical sense to the patient.^26^, ^27^, ^28^ When SDM occurs, patients report higher satisfaction with care decisions and more positive care experiences.^29^, ^30^, ^31^ When done well, SDM epitomizes information exchange and patient involvement. However, despite being evidence‐supported and highly recommended, SDM remains underutilized, with only 42% of Canadian patients reporting an SDM experience and only 21% reporting engagement matching their preference^25^—a finding that is corroborated by our work from multiple patient and clinician perspectives.

Primary care has seen remarkable investment and reorganization ensuring patients can access a multidisciplinary care team and expanded medical services provided within their ‘medical home’. This organizational change has created more PCC delivery,^32^ but as our study illustrates, this care structure does not guarantee collaborative care experiences. Unfortunately, PCC appears to conceptualize the patient as someone who needs to be educated, who must do more work to self‐manage their conditions, and so forth, which effectively operationalizes a degree of paternalism in care delivery and diminishes the patient. While attitudes towards SDM are generally favourable,^33^ across medical disciplines SDM knowledge of the impact on decision outcomes, the patient's role in decision‐making, and a misunderstanding of SDM's time intensity are identified barriers to application in practice. Our study illustrates this knowledge‐practice gap in, for example, the nonalignment of ‘patient information’ and ‘patient involvement’ dimensions. Patients want more equitable care, which we might find in relationship‐centred and strength‐based approaches that honour the lived experiences of the patients and respect them as equal partners in their care, and approaches beyond PCC may facilitate this.

Clinicians also need better training to improve the implementation of collaborative care practices like SDM. Medical students were surveyed in four countries on SDM knowledge, ability to communicate risk and SDM attitudes, revealing SDM as a highly‐trainable skill, however not routinely provided (i.e., the proportion of students receiving SDM training varies from 2% to 74%).^34^ The literature also suggests that patient characteristics may drive clinician SDM engagement decisions. Older, racialized or female patients are less likely to experience SDM despite often strongly desiring engagement in their care decisions.^35^

### 4.1 Limitations

The research team blinded themselves to clinician professions when selecting quotes, in an effort to prevent any bias. However, we acknowledge that this blinding to clinician status (as, e.g., a physician, specialist or nurse) makes the assumption that individuals from these different professions would speak to these issues similarly, which may not have been the case. There may well be differences in perspective by professional groups, but this was not explored in this analysis. This work was conducted within a single provincial health region and may not reflect PCC experiences elsewhere. Nonetheless, clinicians trained outside this region practice here, suggesting SDM training can be universally improved. We endeavoured for diversity, with representation from a range of urban and rural clinical settings and chronic conditions, however, most participants identified as Caucasian with access to study participation opportunities. It seems to be a paradox of not hearing from the people who need equity‐focused approaches the most, as these tend to be the people with the least trust in the establishments and the least capacity to participate in co‐design activities—something that the research team is currently exploring. Finally, participants were asked about care experiences (patients) or how clinicians involve patients in their care without reference to SDM. Thus, conclusions drawn about the patient involvement variability may be biased. Concurrently, without priming study participants to SDM concepts allowed for unbiased and naturally shared experiences without assumptions or reference to specific care models—experienced or not. This work was also conducted in a prepandemic context, so we may not have captured new attitudes resulting from the patient and provider care or SDM experiences that may have shifted with the pandemic. Still, our work helps draw attention to challenging elements of PCC that may persist with the wider adoption of virtual care as a more durable or patient‐centred way of interacting that might create the best opportunity to revisit and embed principles of SDM and PCC.

## 5 CONCLUSIONS

Patient care has recently been restructured to be patient‐centred, however, care collaboration where patients feel respectfully engaged in their health is lacking. This study, which is part one part of a larger research, design and development project that uses a Human‐Centred Design approach to create digital supports for patients with (multiple) chronic conditions in ways that support both PCC and SDM, examined PCC dimension alignment between patient and clinician perspectives. Clinicians may lack insight into the patient perspective on this. Recognizing and responding to patient needs should be foundational in PCC with continued work required to ensure patients are meaningfully engaged. While SDM has the potential to provide a patient–clinician collaboration framework, it remains underutilized despite interventions to support integration into standard care practices. As researchers, we have the opportunity to intentionally push for more collaborative care practices and better education for our clinicians.

## AUTHOR CONTRIBUTIONS

Julie Babione coordinated and conducted most components of the research project including data collection, conducting clinician interviews and focus groups, coordinating transcription and preliminary data analysis, co‐conducting the alignment analysis, manuscript writing and coordinating internal revisions. Dilshaan Panjwani coordinated and co‐conducted clinician interview and focus group sessions, co‐conducted the alignment analysis and participated in manuscript writing and revisions. Sydney Murphy conducted some transcription, co‐conducted the preliminary data analysis and contributed to manuscript writing and revisions. Jessica Van Dyke co‐conducted the preliminary data analysis and contributed to manuscript writing and revisions. Maria Santana contributed to the project's conception and early guidance and contributed to manuscript writing and revisions. Jaime Kaufman oversaw the project and contributed to all stages of the project, as well as manuscript revisions. Peter Sargious contributed to the project's conception, guided the project and contributed to manuscript revisions. Doreen Rabi provided regular project guidance, including data analysis and alignment analysis guidance. She also guided the manuscript's conception, outline and wrote key components of the manuscript discussion. All authors reviewed and approved the final version of the manuscript before submission.

## CONFLICT OF INTEREST

The authors declare no conflict of interest.

## ETHICS STATEMENT

Ethical approval was obtained from the University of Calgary's Conjoint Health Research Ethics Board (REB#13‐1081 and REB#14‐0747).

## Data Availability

The data that support the findings as reported here are available upon request from the corresponding author, Julie Babione and Doreen Rabi. The data are not publicly available due to privacy restrictions, as the data may contain information that could compromise research participant privacy.
